# Causal Impacts of Psychiatric Disorders on Cognition and the Mediating Effect of Oxidative Stress: A Mendelian Randomization Study

**DOI:** 10.3390/antiox14020162

**Published:** 2025-01-29

**Authors:** Yan Gao, Dandan Wang, Qian Wang, Jinfeng Wang, Shuhui Li, Tianqi Wang, Xiaowen Hu, Chunling Wan

**Affiliations:** Bio-X Institutes, Key Laboratory for The Genetics of Developmental and Neuropsychiatric Disorders, Shanghai Jiao Tong University, Shanghai 200030, China; gao_yan@sjtu.edu.cn (Y.G.); wangdandan26@126.com (D.W.); is_wangq@163.com (Q.W.); wangjinfeng@sjtu.edu.cn (J.W.); lishuhui_0929@163.com (S.L.); tianqi-wang@sjtu.edu.cn (T.W.)

**Keywords:** oxidative stress, cognition, psychiatric disorders

## Abstract

Many psychiatric disorders are associated with major cognitive deficits. However, it is uncertain whether these deficits develop as a result of psychiatric disorders and what shared risk factors might mediate this relationship. Here, we utilized the Mendelian randomization (MR) analysis to investigate the complex causal relationship between nine major psychiatric disorders and three cognitive phenotypes, while also examining the potential mediating role of oxidative stress as a shared biological underpinning. Schizophrenia (SZ), major depressive disorder (MDD), and attention deficit hyperactivity disorder (ADHD) showed a decreasing effect on cognitive performance, intelligence, and education, while bipolar disorder (BPD) increased educational attainment. MR-Clust results exhibit the shared genetic basis between SZ and other psychiatric disorders in relation to cognitive function. Furthermore, when oxidative stress was considered as a potential mediating factor, the associations between SZ and the three dimensions of cognition, as well as between MDD and intelligence and ADHD and intelligence, exhibited larger effect sizes than the overall. Mediation MR analysis also supported the causal effects between psychiatric disorders and cognition via oxidative stress traits, including carotene, vitamin E, bilirubin, and uric acid. Finally, summary-based MR identified 29 potential causal associations of oxidative stress genes with both cognitive performance and psychiatric disorders. Our findings highlight the importance of considering oxidative stress in understanding and potentially treating cognitive impairments associated with psychiatric conditions.

## 1. Introduction

Cognitive deficits have become an important focus in major psychiatric disorders, even during periods of remission. They are a core feature of schizophrenia (SZ), and other psychiatrically disordered patients may also exhibit cognitive impairments in comparison to a control population [1,2]. For instance, memory is impaired in most psychiatric disorders: major depressive disorder (MDD) causes difficulties in making decisions and initiating activities [3]; bipolar disorder (BPD) undermines an individual’s ability to inhibit poor responses [4]; and SZ results in a deficit of all the functions listed above [2,5]. These deficits are key predictors of long-term functional and social outcomes and are difficult to treat with current pharmaceutical options or behavioral interventions [6].

The causal relationship between psychiatric disorders and cognition is complex. Prior research has demonstrated that cognitive deficits often precede the onset of psychiatric disorders [2,7], and the cognitive performance of the first-degree relatives of patients tends to be poorer than that observed in the general healthy population [2,7,8,9]. Meanwhile, longitudinal studies have predominantly reported that cognitive function either exhibits a progressive decline or remains stable during the initial episode of the disorders [10,11,12]. This may be either through direct effects on cognitive systems or indirectly through environmental effects, such as poor diet, social deprivation, and so on [13]. Clarifying the causality between psychiatric disorders and cognitive impairments is crucial for a comprehensive understanding of the underlying mechanisms.

Reactive oxygen species are essential for neuronal function and are involved in several cellular processes; however, they become harmful when generated excessively. Oxidative stress has emerged as a “central hub” in the pathophysiology of SZ, which may result from the developmental dysfunction in neuronal signaling, neuroinflammation, and mitochondrial function as well as altering microcircuits and myelinated macrocircuits [14]. Growing epidemiological evidence has shown the close associations between other psychiatric disorders except SZ and redox balance dysregulation, with disturbed oxidative activities and the impairment of antioxidant defensive mechanisms [15,16,17]. Recent studies have highlighted the potential therapeutic benefits of antioxidant treatment in SZ, MDD, and Alzheimer’s disease (AD), with only some focusing on its efficacy in mitigating cognitive dysfunction [18,19]. Moreover, traditional observational studies are often limited by sample size and are susceptible to confounding factors, which may lead to misleading inferences about causal relationships [20,21]. Therefore, it remains unclear whether oxidative stress potentially mediates this association between psychiatric disorders and cognition.

Here, we employed Mendelian randomization (MR) methods to investigate whether there is a causal relationship between psychiatric disorders and cognition, as well as to determine whether oxidative stress serves as a common mediating pathway. This may potentially offer encouraging prospects for targeting the imbalance of oxidative stress to treat or intervene in cognitive impairments associated with psychiatric disorders.

## 2. Materials and Methods

### 2.1. Data Sources

For this study, data sources were derived based on summary-level data conducted primarily from individuals of European ancestry.

For the exposure data, we accessed the summary data of nine major psychiatric disorders available from the Psychiatric Genetics Consortium (PGC), including SZ [22], MDD [23], BPD [24], attention deficit hyperactivity disorder (ADHD) [25], obsessive–compulsive disorder (OCD) [26], autism spectrum disorder (ASD) [27], anorexia nervosa (AN) [28], post-traumatic stress disorder (PTSD) [29], and anxiety disorder (AXD) [30]. These data were the most recent and largest genome-wide association study (GWAS) available at the time of writing (2024/09). The details of these GWASs have been described previously and summarized in Table S1.

Given the complexity of cognitive function, we utilized three global dimensions to characterize cognitive phenotypes: cognitive performance, intelligence, and educational attainment. The GWAS data for cognitive performance used in this study are from a meta-analysis (*n* = 257,841) of published results from the Cognitive Genomics Consortium and UK Biobank [31]. The GWAS data for intelligence are from a meta-analysis of neurocognitive tests (primarily gauging the fluid domains of cognitive functioning), assessing intelligence in 269,867 European individuals [32]. The GWAS data for educational attainment are from a GWAS assessing years of schooling in 307,897 individuals of European ancestry from the UK Biobank [33]. These GWAS data were extracted from the IEU OpenGWAS project (accessed on 1 May 2024 under the IDs: ebi-a-GCST006572, ebi-a-GCST006250, ukb-b-6134). The cohort details and study design are available in the original publication of their GWAS data.

Oxidative stress-related genes were selected as previous studies [34,35,36]. Briefly, these genes were extracted from the GeneCards database using the search term “oxidative stress” and were among the top 10% with the highest relevance scores (Table S2). Genetic predictors for seven oxidative stress injury biomarkers were gained from the IEU OpenGWAS database, including carotene, tocopherol, ascorbate, retinol, albumin, bilirubin, and uric acid (accessed on 1 May 2024 under the IDs: ukb-b-16202, ukb-b-6888, ukb-b-19390, ukb-b-17406, ebi-a-GCST90018945, ebi-a-GCST90018973, and ebi-a-GCST90018977). The GWAS data for albumin, bilirubin, and uric acid were extracted from a meta-analysis of 220 human phenotypes in European ancestry individuals from the UK Biobank and FinnGen (n_total_ = 628,000) [37]. The GWAS data for carotene, tocopherol, ascorbate, and retinol were performed by Ben Elsworth et al. on UK Biobank data [33].

The brain expression quantitative trait locus (eQTL) summary data were derived from a meta-analysis of RNA-seq data, which included 2865 brain cortex tissue samples from 2443 individuals [38]. All individuals were unrelated and of European ancestry. The study focused on single nucleotide polymorphisms (SNPs) located within a 2-megabase (Mb) range from the start and end of each gene.

### 2.2. Statistical Method

#### 2.2.1. Mendelian Randomization Analyses

Mendelian randomization analysis is a powerful tool for establishing causal relationships between exposures and outcomes, utilizing genetic variants as instrumental variables [21,39]. All MR analyses fulfil three core assumptions [21]: (1) genetic variants must be reliably associated with the exposure of interest; (2) genetic variants must not be associated with confounders of the associations; (3) genetic variants should not be independently associated with the outcome through any pathway other than through the exposure (i.e., horizontal pleiotropy).

Two-sample univariable MR analyses were performed to estimate the causal effects of psychiatric disorders on cognition, respectively. For three exposure traits (SZ, MDD, and BPD), the genetic variants were filtered by *p*-value < 5 × 10^−8^. For the other six exposure traits, the stringent threshold of a *p*-value below 5 × 10^−8^ proved insufficient for selecting a robust set of instrumental variables. Consequently, a less stringent *p*-value standard below 5 × 10^−6^ was employed to facilitate the subsequent analysis. Each instrument’s result set of variants was clumped with r2 = 0.001 and clump windows = 10,000 kb in the 1000 Genomes reference panel within European data. The inverse variance weighted (IVW) method was used as the main MR method, which combines the Wald ratio estimates of each SNP into a single causal estimate for each exposure using the random effects meta-analysis approach [40]. Additional MR methods were incorporated for enhanced validity, as described in the Section 2.3 Sensitivity Analyses section.

To further explore the potentially mediated role of oxidative stress in these causal associations, the MR analysis under restricted instruments was applied. The selection of instrumental variables was refined to include only SNPs located within genes of the oxidative stress gene set, as recorded in the Ensembl database. Another stringent standard was set, refining the instrumental variables to SNPs significantly related (*p*-value standard below 5 × 10^−5^) to the genes of the oxidative stress gene set in brain eQTL [38]. Moreover, the analyses assessing whether oxidative stress injury biomarkers act as causal mediators were conducted under the two-step MR framework [41]. The first step was to estimate the causal effect of genetically determined psychiatric disorders on cognition, and the second step was to estimate the causal effect of the mediators on cognition.

All analyses were conducted using R (v4.0.3). MR analyses were performed using the TwoSampleMR R package (v0.6.7) [42].

#### 2.2.2. Summary Data-Based Mendelian Randomization

To provide additional support for the link between oxidative stress and two phenotypes, a summary data-based MR (SMR) analysis [43] was applied using GWAS data from psychiatric disorders, cognition, and brain eQTL.

Similar to MR analysis, only SNP–gene associations with a *p*-value standard below 5 × 10^−8^ were included in the SMR analysis. The significant SMR results could reflect a pleiotropic or linkage model. The heterogeneity in dependent instruments (HEIDI) test was used to demonstrate that genes associated with psychiatric disorders and cognition were not due to genetic linkage. A significant SMR association was defined as *p* < 0.05, while HEIDI *p* > 0.05 indicated that the association was caused by a shared gene variant. To account for multiple tests, the Bonferroni approach was used to adjust *p*-SMR values. These analyses were performed by the SMR software (version 1.3.1) [43].

### 2.3. Sensitivity Analyses

The IVW analysis was used as the standard MR analysis, and the Egger [44] and weight median MR methods [45] were also applied to evaluate the validity of the analyzed genetic instruments and support the causal effects found with IVW.

MR-Egger regression was applied to evaluate the overall horizontal pleiotropy [44]. When the Egger intercept was close to zero (e.g., <0.002), and the *P*-value was large, this could be interpreted as no evidence of a substantial. Cochran’s Q-statistic was applied to evaluate the heterogeneity among the genetic variants [46], with a high Q-statistic accompanied by a small *p*-value indicating potential heterogeneity. MR-PRESSO was another way to evaluate the heterogeneity by evaluating the consistency between the IVW results after the removal of influential outliers and the raw IVW results [47]. It provided an outlier-corrected pleiotropy-robust causal estimate as a result. MR-Egger and Cochran’s Q-statistic analyses were performed using the TwoSampleMR R package (v0.6.7) [42]. MR-PRESSO analyses were performed using the “MR-PRESSO” R package v1.0 [47].

F-statistics analysis was applied to evaluate the instrument strength, with an F-value greater than 10 indicating sufficient strength for minimal weak instrument bias in the analysis [48]. Steiger filtering was applied to evaluate the possibility of reverse causation, which is based on whether each instrument explains more variance (R^2^) in the exposure than in the outcome [42]. Steiger analyses were performed using the TwoSampleMR R package (v0.6.7) [42].

### 2.4. Additional Analyses

#### 2.4.1. MR-Clust

MR-Clust was applied to identify distinct causal pathways linking each psychiatric disorder to cognition. This method calculates the MR causal estimate for each instrument variant and clusters into distinct groups with similar causal estimates by maximizing the likelihood of a mixture of normal distributions. MR-Clust assigns variants to K clusters, where all variants have similar causal ratio estimates, a “null” cluster (variants with a null effect), and a “junk” cluster. An instrument variant is only assigned to a cluster with a great conditional probability of assignment (score > 0.9); if it is lower than this, then the variant is assigned to the junk cluster without further analyses. A cluster may represent a distinct pathway through which exposure is related to the outcome, and investigating heterogeneous estimates in this way may reveal additional information about the exposure–outcome relationship. MR-Clust analyses were performed using the “mrclust” R package v0.1.0 [49].

#### 2.4.2. Phenome-Wide Association Studies

To further examine the instruments of the psychiatric disorders and better understand the potential sources of effect heterogeneity, we performed a phenome-wide association study (PheWAS) analysis [50]. The SNP-associated phenotypes were filtered based on the following criteria: (1) we discarded traits/datasets with *p*-value greater than 5 × 10^−8^; (2) we removed all traits/datasets derived from high-throughput molecular trait panels (trait IDs starting with “eqtl-” and “pqtl-”). These analyses were performed using the “ieugwasr” R package v1.0.1 and exacted the information through the Open-GWAS database [33].

We presented PheWAS analysis for each instrument grouped by clusters determined by the MR-Clust algorithm. This method may help identify distinct causal mechanisms specific to each psychiatric disorder’s impact on cognition, as well as uncovering common causal mechanisms affecting cognition across different psychiatric disorders. We categorized phenotypes significantly associated with SNP into three distinct groups: psychiatric phenotypes, other psychiatric phenotypes, and other phenotypes.

## 3. Results

### 3.1. Study Design

**Figure 1:**
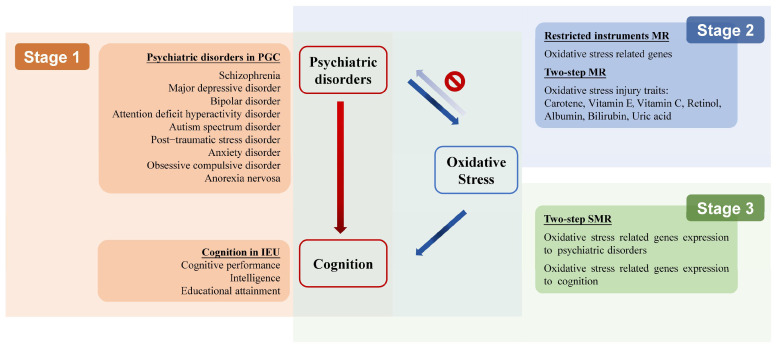
Overview of the study design. PGC, Psychiatric Genetics Consortium; IEU, the MRC Integrative Epidemiology Unit; MR, Mendelian randomization; SMR, summary data-based MR.

This study included three stages of analyses (for study design see Figure 1). In the first stage, we assessed the causal associations between nine psychiatric disorders and cognition using MR, which utilized SNPs as instrumental variables to proxy for each exposure. We further employed sensitivity analysis methods and conducted MR-Clust combined with PheWAS to examine heterogeneous effects, enhancing the understanding of the shared effects on cognition across different psychiatric disorders. In the second stage, only psychiatric disorders that had established a causal association with cognition in the first stage were included. We examined oxidative stress’s potential as a mediator in psychiatric–cognition associations using two-step MR and MR with restricted instrumental variables. In the third stage, we explored the genes whose expression change in the brain may contribute to the susceptibility of psychiatric disorders and cognitive function using a two-step SMR. These common genes could reveal the underlying biological mechanisms and uncover potential therapeutic targets for psychiatric disorders associated with cognitive impairment.

This study is reported according to the strengthening the reporting of observational studies in epidemiology using Mendelian randomization (STROBE-MR) guideline.

### 3.2. Causal Effects of Psychiatric Disorders on Cognition

**Figure 2:**
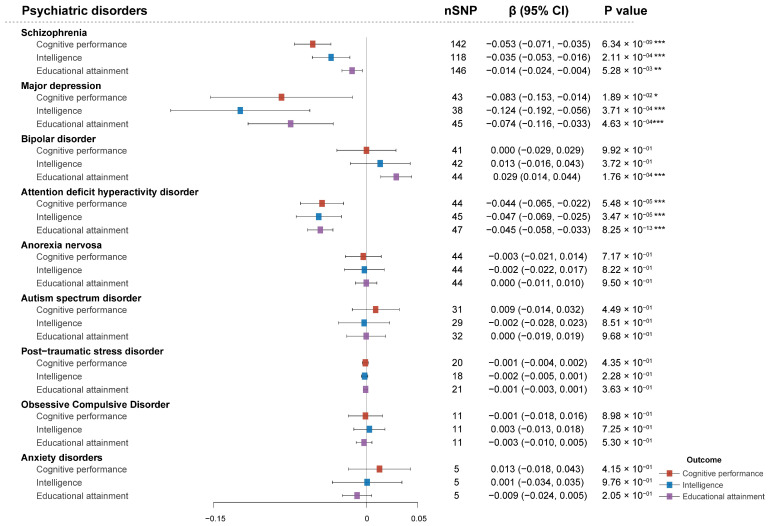
Mendelian randomization (MR) estimates of the causal associations of nine psychiatric disorders with three dimensions of cognitive function. The exposure traits are psychiatric disorders, including schizophrenia (SZ), major depressive disorder (MDD), bipolar disorder (BPD), attention deficit hyperactivity disorder (ADHD), obsessive–compulsive disorder (OCD), autism spectrum disorder (ASD), anorexia nervosa (AN), post-traumatic stress disorder (PTSD), and anxiety disorder (AXD). The outcome traits are three dimensions of cognitive phenotypes, including cognitive performance, intelligence, and educational attainment. The error bars indicate 95% confidence intervals around the point estimate (beta coefficient) from IVW MR analyses. Red plots represent the effect of psychiatric disorders on cognitive performance; blue plots represent the effect of psychiatric disorders on intelligence; purple plots represent the effect of psychiatric disorders on educational attainment. *p < 0.05; **p < 0.01; ***p < 0.001. Source data are included in Table S3.

The univariable MR was used to assess the impact of nine prevalent psychiatric disorders on cognition. Cognitive performance, intelligence, and educational attainment are three different dimensions of global cognition exhibited highly consistently. SZ, MDD, BPD, and ADHD show significantly casual associations with cognitive indexes. The MR results showed that SZ (β: −0.053; [95% CI: −0.071 to −0.035]), MDD (β: −0.083; [95% CI: −0.153 to −0.014]), and ADHD (β: −0.044; [95% CI: −0.065 to −0.022]) are significant contributors to poor cognitive performance (Figure 2 and Table S3). Similarly, SZ, MDD, and ADHD were also found to reduce both intelligence quotient scores and educational attainment (Figure 2 and Table S3). In contrast, BPD was modestly associated with higher educational attainment (β: 0.029; [95% CI: 0.014 to 0.044]). The other psychiatric disorders did not show any causal relationship with any dimension of cognition.

Sensitivity analyses were further conducted for each causal relationship. The MR results from the weighted median method were consistent with those obtained from the main MR analysis using the IVW method (Table S3). Additionally, the MR results from the MR-Egger method did not show any significant associations (Table S3). These results indicated robust causal associations between nine psychiatric disorders and cognition, as determined by the IVW method. The Egger intercept was below 0.01 across all the estimated causal effects of psychiatric disorder measures on cognition, suggesting a lower likelihood of directional horizontal pleiotropy (Table S4). The F-statistics were greater than 10, which did not indicate weak instrument bias, and the Q-statistics did not reveal excessive heterogeneity (Table S4). The outlier-corrected total IVW estimates were similar to the raw IVW result, with estimates for SZ showing particularly strong evidence of an effect on cognition (Table S5).

**Figure 3:**
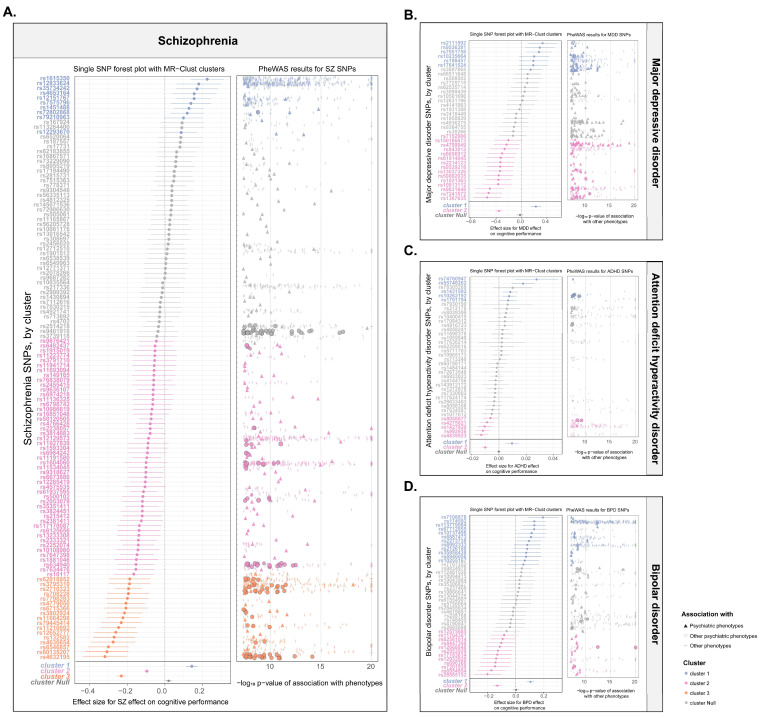
Exploring the heterogeneity of genetic instruments of four psychiatric disorders on cognitive performance. (**A**) Plots of schizophrenia on cognitive performance. (**B**) Plots of major depressive disorder on cognitive performance. (**C**) Plots of attention deficit hyperactivity disorder on cognitive performance. (**D**) Plots of bipolar disorder on cognitive performance. In each panel, the left plot is single SNP forest plot of each psychiatric disorders on cognitive performance. SNPs colored by the cluster membership assigned by MR-Clust. The error bars are 95% confidence intervals of the Wald ratio point estimate (beta coefficient) for each variant. The inverse-variance weighted (IVW) MR estimates for each cluster are presented below single SNP estimates. Right plot is bubble plot of PheWAS results for genetic variants. The data points are other traits associated with SNPs (*y*-axis) at *p*-value (adjusted) < 5 × 10^−8^ (*x*-axis, -log10 scale, capped at value 20). The color shows the cluster membership, in the same palette and order as in the left plot.

MR-Clust clustered the instrumental variants used to estimate causal relationships, with each cluster potentially representing a distinct pathway, through which exposure was related to the outcome. The subsequent cluster analysis of the SZ genetic instruments displayed four distinct clusters, with MR used to quantify the effect of each cluster on cognitive performance (Figure 3A). For Cluster 3 (SNPs *n* = 17) and Cluster 2 (SNPs n = 49), the SNPs were associated with a higher risk of SZ and a decreased cognitive performance (Cluster 3: −0.232 [95% CI −0.257 to −0.207]; Cluster 2: −0.096 [95% CI −0.110 to −0.0826]; Figure 3A). Compared with the overall causal association on cognitive performance, these cluster associations were consistent in direction but exhibited larger effect sizes. In the further PheWAS analysis, many SNPs in these two clusters were also associated with other psychiatric traits (Table S6), suggesting that the SNPs in these clusters shared a common genetic basis across a spectrum of psychiatric traits. Similarly, in Cluster 1 in MDD (Figure 3B) and Cluster 1 in ADHD (Figure 3C), the causal associations, respectively, were consistent with the direction overall (Cluster 2 in MDD: −0.353 [95% CI −0.411 to −0.296]; Cluster 2 in ADHD: −0.010 [95% CI −0.013 to −0.008]), and a multitude of SNPs were also associated with other psychiatric traits (Tables S7 and S8). Although BPD showed an overall enhanced effect on education, the cluster enriched the variants that were associated with other psychiatric traits contributed to poorer cognitive performance (Cluster 2 in BPD: −0.132 [95% CI −0.162 to −0.102]; Figure 3D, Table S9). These results consistently demonstrate the effects of these psychiatric disorders on both intelligence (Figure S1) and educational attainment (Figure S2). These findings indicated that the SNPs within these clusters are more indicative of a broad spectrum of psychiatric disorders, pointing to the potential shared genetic basis underlying the impairing effects of the psychiatric disorders on cognition.

### 3.3. Oxidative Stress as a Mediator of Psychiatric Disorders to Cognitive Impairment

Given that oxidative stress has been widely reported to be closely associated with a variety of psychiatric disorders, we conducted analyses to explore its potential as a shared mediating factor in the causal impact of SZ, MDD, BPD, and ADHD on cognition, as we revealed above. Two analyses were performed: MR analysis with restricted instrumental variables related to oxidative stress genes and a two-step MR analysis focusing on oxidative stress traits.

**Figure 4:**
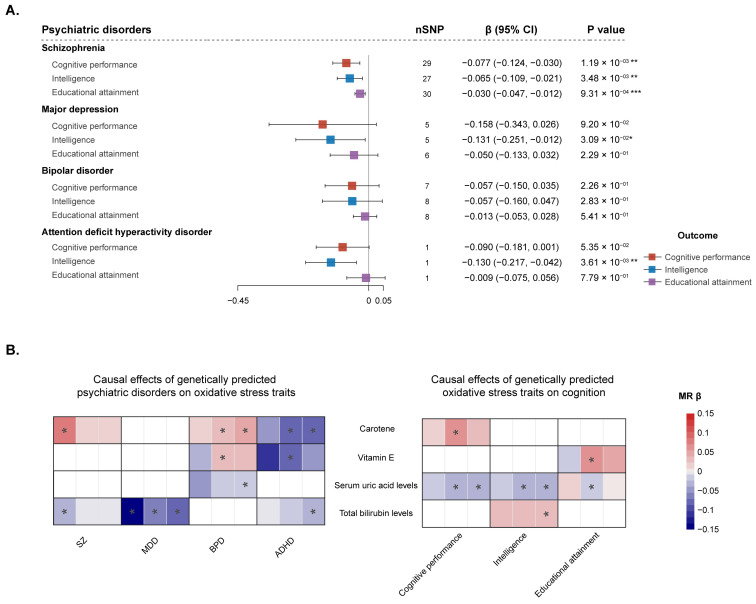
Mendelian randomization (MR) estimates of the mediation by oxidative stress in the causal association between psychiatric disorders and cognitive function. (**A**) The estimates of MR analysis using restricted instrumental variables within oxidative stress genes. The exposure traits are psychiatric disorders, including schizophrenia (SZ), major depressive disorder (MDD), bipolar disorder (BPD), and attention deficit hyperactivity disorder (ADHD). The outcome traits are three dimensions of cognitive phenotypes, including cognitive performance, intelligence, and educational attainment. The error bars indicate 95% confidence intervals around the point estimate (beta coefficient) from IVW MR analyses. Red plots represent the effect of psychiatric disorders on cognitive performance; blue plots represent the effect of psychiatric disorders on intelligence; purple plots represent the effect of psychiatric disorders on educational attainment. (**B**) Results of the two-step MR analysis focusing on oxidative stress traits. The left plot shows the causal effects of psychiatric disorders (exposure traits) on oxidative stress traits (outcome traits). The right plot shows the causal effects of oxidative stress traits (exposure traits) on cognition (outcome traits). Colored cells indicate the point estimate (beta coefficient) from MR analyses, including MR-Egger analysis (left), IVW MR analysis (median), and weight median MR analysis (right). * *p* < 0.05; ** *p* < 0.01; *** *p* < 0.001.

In the MR analysis with restricted instrumental variables, the SNPs used for MR causal analysis were restricted to those located in genes related to oxidative stress (Tables S2 and S10). The restricted causal effects of SZ, MDD, and ADHD on cognition still showed similarities with the overall effects (Figure 4A and Table S11), indicating that oxidative stress within the context of these disorders may mediately lead to poor cognitive performance, low intelligence scores, and low educational attainment. Moreover, it is important to note that the estimates in these analyses were of a larger magnitude than those overall (Figure 2 and Figure 4A), which may imply the presence of heterogeneity within the overall estimates. Meanwhile, under the restricted condition considering oxidative stress, BPD did not show a significant contribution to better education as the overall estimate, as indicated in Figure 2, but instead showed a tendency to contribute to poorer cognitive performance (Figure 4A and Table S11). When the instrumental variants were further restricted to SNPs that can affect the expression of oxidative stress-related genes in the brain (Tables S2 and S12), the results also revealed that SZ, MDD, and ADHD were causally associated with poor cognitive function under this condition of oxidative stress (Figure S3 and Table S11). Sensitivity analyses confirmed these IVW estimates and suggested that they are less likely to be driven by horizontal pleiotropy (Tables S13 and S14). These findings suggest that SZ, MDD, and ADHD may amplify cognitive impairments, with oxidative stress potentially acting as a mediating factor. Given that cognitive function slightly influences the risk of SZ and ADHD (Table S15), the reverse MR analysis was conducted to explore whether cognitive function increases the risk of psychiatric disorders when oxidative stress is considered to be a mediating factor. The results indicated that cognitive damage did not significantly affect psychiatric disorders under the restricted conditions of oxidative stress (Table S15).

In the two-step MR analyses focusing on oxidative stress traits, psychiatric disorders were found to be causally associated with several traits (Figure 4B and Table S16). Those results showed that the risk of SZ, MDD, and ADHD was associated with decreased levels of carotene, vitamin C, vitamin E, and bilirubin. Similarly, BPD risk was shown to increase the levels of vitamin E and carotene, which is in the opposite direction of the effects observed with other psychiatric disorders. Meanwhile, only carotene, vitamin E, uric acid, and bilirubin showed causal associations with cognition (Figure 4B), which implies that these oxidative stress traits may convey the causal effects from psychiatric disorders to cognitive function. Furthermore, there was limited evidence of a causal effect in the opposite direction, suggesting that these traits do not significantly influence the development of psychiatric disorders (Figure S4 and Table S16). These causal effects were verified by additional sensitivity analyses (Table S17). These results conclusively identify oxidative stress traits as a potential shared mediator in the intricate relationship linking psychiatric disorders, such as SZ, MDD, BPD, and ADHD to cognitive deficits.

### 3.4. Oxidative Stress-Related Genes Involved in Psychiatric Disorders–Cognition Causal Relationships

Considering that oxidative stress may play a role in the cognitive impairment associated with psychiatric disorders, we then hypothesized that gene expression in the brain may explain the plausible causality. Therefore, we aimed to identify candidate causal oxidative stress genes in both cognition and psychiatric disorders. Using the SMR statistical inference method, 97 oxidative stress genes with little evidence of heterogeneity P_HEIDI_ ≥ 0.05 may affect cognitive performance (Table S18). Similar analyses were conducted in SZ, BPD, MDD, and ADHD, and the results showed that 95, 81, 77, and 44 gene–trait associations in the brain significantly are, respectively, related to risk for these psychiatric disorders followed by the HEIDI test (P_HEIDI_ ≥ 0.05) implemented (Tables S19–S22). The estimates of genes in the opposite direction were enrolled in further analyses, which indicated that the genes with a higher risk of disorder showed poorer cognitive function. Among these, 15 association signals for SZ, 10 for MDD, 8 for BPD, and 5 for ADHD were included in the genes that contributed to cognitive performance and exhibited estimates (Table 1). The genes *BCHE* and *CCS* were implicated in multiple psychiatric disorder–cognition associations. The lower mRNA levels of *BCHE* and *CCS* were significantly associated with worse cognitive performance as well as an increased genetic risk of SZ, MDD, and BPD. In summary, our comprehensive analysis identified a set of oxidative stress genes that are not only associated with cognitive performance but also linked to the risk of multiple psychiatric disorders, highlighting the potential role of the common genetic basis shared among a spectrum of psychiatric traits with cognitive deficits.

## 4. Discussion

Our study aimed to identify the influence of prevalent psychiatric disorders on cognition and to determine whether oxidative stress plays a shared mediating role in cognitive impairment. Using the MR method, we explored the associations between nine prevalent psychiatric disorders and three cognitive phenotypes to gain insights into their causal effects. Only SZ, MDD, and ADHD showed a potential impairment in cognitive function; whereas, BPD may promote educational attainment. However, when considering the mediating role of oxidative stress, all above four psychiatric disorders demonstrated a detrimental effect on cognition. Further analysis revealed several shared genes underlying psychiatric disorders that could also influence cognition, which may inform the development of novel and effective transdiagnostic cognitive treatments.

Many studies over the past decades have posited a vicious cycle between cognitive decline and the risk of psychiatric disorders. Cognitive impairments are not only evident during the first episode of psychiatric disorders but also persist as ongoing damage following the onset of the disease [1]. Our results conducted by MR analyses provided evidence for the progressive decline in cognitive function over the course of several psychiatric disorders. A previous study has found that individuals at clinical high risk (CHR) for psychosis displayed worse cognitive function compared to CHR individuals who did not transition [51]. Furthermore, long-term longitudinal studies spanning over 10 years in duration have demonstrated a significant decline in cognitive function in individuals with psychiatric disorders and a more rapid decline compared with normal ageing [10,11,12]. SZ is characterized by a defining feature of a decline in cognitive function over the course of the disease [52], which includes a broad pattern of cognitive deficits ranging from attention and working memory to social cognition and language [2,53,54]. MDD and BPD also recognized neurocognitive impairment as one of the diagnosis criteria [55,56]. ADHD showed a strong association with cognitive performance, especially with educational outcomes, in genetic liability studies and observational research [57]. Our findings concur with recent studies that indicate a limited connection between various psychiatric disorders, such as anorexia nervosa, autism spectrum disorder, post-traumatic stress disorder, obsessive–compulsive disorder, anxiety disorders, and cognitive function [1].

Our sensitivity results in MR analyses revealed that the genetic variants used showed heterogeneous estimates, which is consistent with the complexity of the relationship between psychiatric disorders and cognition. The MR clustering method decomposes heterogeneity into different clusters. For SZ and cognitive performance, the majority of instrumental variables, which formed strong effect clusters that predict impaired cognitive function, were also associated with other psychiatric disorders and psychiatric traits. Consistent with this, many identified risk alleles for SZ were associated with poor cognitive performance [22,58,59]. Similarly, the polygenic risk score for BPD correlates with diminished cognitive function, primarily due to shared genetic variants with SZ [60,61]. The BPD-specific risk alleles are associated with better cognitive performance [62]. These results indicate that the relationship between psychiatric disorders and cognition is characterized by heterogeneity, with different psychiatric disorders potentially sharing an underlying genetic basis that could impair cognitive function.

Another noteworthy finding of this study is the identification of the shared mediating roles of oxidative stress in the association between several psychiatric disorders—SZ, MDD, BPD, and ADHD—and cognition. Specifically, SZ, MDD, and ADHD were found to contribute to cognitive impairment under the oxidative stress mediating. Meanwhile, while BPD appears to exert a protective effect on cognitive function at an overall level, it tends to be associated with poorer cognitive performance under oxidative stress mediating. The associations between redox imbalance and cognitive disruption have been extensively studied in the context of aging and neurodegenerative diseases, such as AD and mild cognitive impairment. The role of oxidative stress in the relationship between psychiatric disorders and cognition remains largely controversial. Clinical observation studies have observed disturbed levels of oxidative stress traits, such as increased levels of peroxides and reduced activity of antioxidant enzymes in patients with SZ, MDD, BPD and ADHD [14,15,16,17], with several studies also associated with cognitive performance [63,64,65]. Previously, a bidirectional MR study suggested that genetically predicted MDD was significantly associated with decreased total bilirubin, and ADHD was significantly associated with decreased ascorbate [66]. Our MR analysis revealed that redox dysregulation may serve as a key factor in the causal effects of psychiatric disorders on cognition. Recently, there has been a growing consensus that oxidative stress may serve as a central hub in the pathophysiology of SZ [14,67]. This hypothesis points out that the developmental impairments in NMDAR-mediated signaling, neuroinflammation, and mitochondrial function may initiate the redox dysregulation in SZ. The redox dysregulation may subsequently lead to neuronal abnormalities, ultimately manifesting as cognitive and psychiatric symptoms. Given that redox dysregulation was also observed in SZ, MDD, BPD, and ADHD [68], it may be the shared pathway to neuronal abnormalities and cognitive impairment in those diseases. This may provide robust evidence supporting the shared pathogenic mechanisms and potential therapeutic directions for these psychiatric disorders. Furthermore, we investigated whether the reverse impact of cognitive function on psychiatric disorders is similarly mediated by redox dysregulation, but our findings did not substantiate this hypothesis. This suggested that the occurrence of cognitive impairments may not be involved in the disease process by affecting oxidative stress levels, as it does in the other direction.

Upon the establishment of the causal impacts of the four identified psychiatric disorders on cognition and the mediating effect of oxidative stress, we further identified a set of causal oxidative stress-related genes underlying both cognition and psychiatric disorders, with the aim to provide potential therapeutic targets. BCHE and CCS are conspicuous in Table 1, as these two genes present causal associations prevalent with three psychiatric disorders except for ADHD and cognition. BChE is an enzyme that inactivates the neurotransmitter acetylcholine by catalyzing its hydrolysis to choline and acetic acid, similar to its paralog AChE. BChE does not have a direct relationship with oxidative stress in terms of biological function. However, numerous studies have indicated that inhibiting BChE can enhance antioxidant properties, thereby exerting a neuroprotective effect in AD mice models [69,70]. Considering the role of AChE and BChE in suppressing neurotransmitter acetylcholine levels, numerous studies have demonstrated its potential as a therapeutic target for alleviating cognitive impairments in AD and SZ [71,72]. However, due to the limitations of AChE inhibitors [73,74], including dosage constraints and inadequate long-term efficacy, BChE inhibitors are emerging as a new therapeutic target, suggesting that inhibiting BChE could be a potential therapeutic approach for cognitive impairments in SZ, BPD, and MDD. CCS is a copper chaperone that specifically delivers Cu to copper/zinc superoxide dismutase (SOD1), an antioxidant protein found in the mitochondrial intermembrane space and cytoplasm. By potentially activating SOD1, CCS plays a crucial role in redox regulation. The abnormalities in the CCS level may lead to impaired copper metabolism inducing apoptosis, paraptosis, pyroptosis, ferroptosis, and cuproptosis [75]. Wilson’s disease and Menkes syndrome are copper metabolism-related disorders that exhibit neurocognitive impairments; Wilson’s disease can manifest symptoms with MDD, BPD, and personality changes [76]. It is worth noting that these two genes do not demonstrate a potential influence on cognitive functioning in ADHD, indicating that, while cognitive impairments in ADHD are mediated by oxidative stress, the genetic basis involved may be distinct. Other oxidative stress-related genes, as we proposed in Table 1, are functionally involved in biological processes, such as antioxidant defense (*GSR, SP1*), mitochondrial homeostasis and energy metabolism (*MAP1LC3B, NDUFA2, TRIT1, GATB, SPG7, FARS2, PRORP, TUFM),* glucose and lipid metabolism *(RAF1, TKT, PTGIS, ACOX1, ACOX2*), immune regulation (*PPIA, TRAF3, GRN, STK4, PARP1, OLR1*), cytoskeleton organization (*ACTB, PAK1*), etc. The significant role of oxidative stress in the pathophysiology of these conditions suggests that the genes reported in this study hold a promise as powerful potential therapeutic targets.

### Limitations

There are several potential limitations to this study. First, the GWAS and eQTL studies utilized were based on populations of European ancestry. Although genetic associations with most psychiatric disorders that are linked to explicit biological processes may be consistent across populations of different genetic ancestries [77], the genetic architecture of cognition and oxidative stress traits has not been thoroughly investigated in non-European ancestry populations. Similarly, although sex and gender are known to influence psychiatric disorders, oxidative stress, and cognitive performance [78,79,80], the lack of stratified data in the GWAS summary data for cognition and oxidative stress precluded analysis in this study. Second, although we focused on the most prevalent and important cognitive function indexes as potential outcomes, the causal effects of psychiatric disorders on cognitive function may not be fully explained in this study due to the complexity of cognitive performance. For example, the early onset of ASD suggests that educational attainment may not comprehensively represent an individual’s cognitive abilities. Third, our MR analyses in stage 2 were restricted to oxidative stress injury biomarkers that had been measured and had genome-wide significant loci. As a result, some classic markers of oxidative stress damage, such as 8-hydroxy-2-deoxyguanosine (8-OHdG) and malondialdehyde (MDA), were excluded from our analysis due to the lack of corresponding GWAS data. Hence, we performed the restricted gene MR analyses to collectively support the mediating role of oxidative stress in the causal effects of psychiatric disorders on cognition. Fourth, the MR analysis assumes a linear relationship between exposure and outcome. However, it should be noted that the relationship between antioxidant levels and cognitive function may be complex and not always linear [81,82]. Fifth, considering the close association between psychiatric disorders, cognition, and brain function, this study utilized brain-specific eQTLs for the SMR analysis. However, due to the complexity of overall brain function, eQTL data from specific brain regions may be more representative of cognitive performance and psychiatric disorders.

## 5. Conclusions

Our study focused on the causal associations among multiple psychiatric disorders, cognitive function, and the potential mediating role of oxidative stress. Our study provided evidence supporting the progressive decline in cognitive function following the onset of SZ, MDD, BPD, and ADHD. The decline in cognition across these different psychiatric disorders may share a common biological basis in oxidative stress. Notably, two genes, *BCHE* and *CCS*, along with biomarkers such as carotene, vitamin E, and bilirubin, demonstrate a significant relationship between psychiatric disorders and cognitive impairment. These findings suggest that targeting redox dysregulation could be a promising therapeutic strategy for addressing cognitive deficits in psychiatric disorders.

These insights not only deepen our comprehension of the mechanisms underlying cognitive impairments in psychiatric disorders but also inform the development of innovative therapeutic interventions.

## Figures and Tables

**Table 1:** Summary data-based Mendelian randomization results for the associations between cognitive performance, psychiatric disorders risk, and oxidative stress-related gene expression.

Gene Symbol	Ensembl ID	Chr	Os_Relevance score	Cognitive Performance				Psychiatric Disorders				
				b_SMR	se_SMR	p_SMR	p_HEIDI	Disorders	b_SMR	se_SMR	p_SMR	p_HEIDI
GSR	ENSG00000104687	8	40.98	−0.025	0.011	0.022	0.189	SZ	0.070	0.034	0.038	0.919
TUFM	ENSG00000178952	16	33.35	−0.059	0.008	0	0.928		0.053	0.021	0.012	0.988
PARP1	ENSG00000143799	1	26.11	−0.039	0.014	0.005	0.753		0.093	0.04	0.02	0.226
GATB	ENSG00000059691	4	17.64	0.03	0.006	0	0.064		−0.033	0.017	0.049	0.2
ACOX1	ENSG00000161533	17	17.41	0.022	0.011	0.038	0.987		−0.085	0.034	0.013	0.903
SP1	ENSG00000185591	12	17.02	0.044	0.014	0.002	0.196		−0.149	0.044	0.001	0.333
PTGIS	ENSG00000124212	20	11.88	−0.021	0.009	0.023	0.127		0.056	0.028	0.047	0.119
BCHE	ENSG00000114200	3	10.37	−0.014	0.005	0.009	0.772		0.040	0.016	0.01	0.283
PPIA	ENSG00000196262	7	9.75	−0.034	0.014	0.013	0.347		0.114	0.043	0.008	0.068
ACTB	ENSG00000075624	7	8.74	−0.037	0.015	0.015	0.447		0.125	0.047	0.008	0.121
GRN	ENSG00000030582	17	8.02	−0.014	0.006	0.016	0.262		0.041	0.018	0.026	0.403
CCS	ENSG00000173992	11	7.18	−0.022	0.01	0.036	0.601		0.063	0.031	0.047	0.239
TRAF3	ENSG00000131323	14	7.06	−0.025	0.008	0.002	0.256		0.054	0.024	0.023	0.078
ABCC3	ENSG00000108846	17	6.63	0.017	0.006	0.006	0.769		−0.045	0.019	0.019	0.386
MAP1LC3B	ENSG00000140941	16	6.63	0.046	0.015	0.002	0.908		−0.086	0.042	0.04	0.541
OLR1	ENSG00000173391	12	31.98	0.026	0.011	0.017	0.596	MDD	−0.033	0.016	0.039	0.436
TRIT1	ENSG00000043514	1	20.01	0.009	0.004	0.032	0.23		−0.015	0.007	0.024	0.086
PRORP	ENSG00000100890	14	16.23	−0.017	0.009	0.042	0.061		0.037	0.013	0.005	0.902
EGFR	ENSG00000146648	7	14.54	−0.013	0.005	0.005	0.615		0.016	0.007	0.021	0.47
STK4	ENSG00000101109	20	11.4	0.026	0.01	0.005	0.665		−0.027	0.014	0.05	0.652
BCHE	ENSG00000114200	3	10.37	−0.014	0.005	0.009	0.772		0.025	0.008	0.002	0.041
ACTB	ENSG00000075624	7	8.74	−0.037	0.015	0.015	0.447		0.056	0.023	0.015	0.273
CCS	ENSG00000173992	11	7.18	−0.022	0.01	0.036	0.601		0.071	0.017	0	0.35
TRAF3	ENSG00000131323	14	7.06	−0.025	0.008	0.002	0.256		0.034	0.012	0.005	0.013
TKT	ENSG00000163931	3	6.85	−0.018	0.007	0.013	0.149		0.038	0.011	0.001	0.33
GATB	ENSG00000059691	4	17.64	0.03	0.006	0	0.064	BPD	−0.045	0.018	0.012	0.954
RAF1	ENSG00000132155	3	11.7	0.062	0.018	0	0.129		−0.124	0.053	0.019	0.1
BCHE	ENSG00000114200	3	10.37	−0.014	0.005	0.009	0.772		0.047	0.017	0.006	0.197
NDUFA2	ENSG00000131495	5	10.03	0.041	0.008	0	0.212		−0.079	0.025	0.001	0.067
PPIA	ENSG00000196262	7	9.75	−0.034	0.014	0.013	0.347		0.11	0.045	0.015	0.138
RPS6KB1	ENSG00000108443	17	7.85	−0.03	0.012	0.013	0.094		0.084	0.039	0.031	0.099
CCS	ENSG00000173992	11	7.18	−0.022	0.01	0.036	0.601		0.141	0.038	0	0.067
SPG7	ENSG00000197912	16	6.76	−0.012	0.005	0.016	0.325		0.042	0.016	0.01	0.109
ABCC3	ENSG00000108846	17	6.63	0.017	0.006	0.006	0.769		−0.059	0.022	0.006	0.45
FARS2	ENSG00000145982	6	55.1	−0.02	0.007	0.007	0.075	ADHD	0.067	0.034	0.05	0.481
TRIT1	ENSG00000043514	1	20.01	0.009	0.004	0.032	0.23		−0.054	0.022	0.013	0.187
CTNNB1	ENSG00000168036	3	12.24	0.03	0.006	0	0.628		−0.098	0.027	0	0.583
ACOX2	ENSG00000168306	3	7.74	0.032	0.011	0.004	0.78		−0.167	0.065	0.01	0.956
PAK1	ENSG00000149269	11	7.59	0.021	0.01	0.039	0.13		−0.106	0.047	0.026	0.329

Note: Chr, chromosome; Os_Relevance score, the relevance score of oxidative stress-related genes reported in GeneCards database; SMR, summary data-based Mendelian randomization; SZ, schizophrenia; MDD, major depressive disorder; BPD, bipolar disorder; ADHD, attention deficit hyperactivity disorder.

## Data Availability

This study was based on publicly available summarized data (IEU OPEN GWAS PROJECT: https://gwas.mrcieu.ac.uk/ (accessed on 1 May 2024)), PGC (https://www.med.unc.edu/pgc/ (accessed on 1 May 2024)), and SMR (https://yanglab.westlake.edu.cn/software/smr/ (accessed on 1 May 2024)). All data and codes reported in this paper will be shared by the lead contact upon request.

## Supplementary Materials

The following supporting information can be downloaded at: https://www.mdpi.com/article/10.3390/antiox14020162/s1, Figure S1. Exploring the heterogeneity of genetic instruments of four psychiatric disorders on intelligence; Figure S2. Exploring the heterogeneity of genetic instruments of four psychiatric disorders on educational attainment; Figure S3. The estimates of Mendelian randomization analysis with restricted instrumental variables associated with the brain expression of oxidative stress genes; Figure S4. Results of Mendelian randomization analysis focusing on the causal effects of oxidative stress traits on psychiatric disorders; Table S1. Summary of the GWAS data used in the MR analyses; Table S2. List of oxidative stress-related genes; Table S3. Univariable MR of psychiatric disorders on cognition; Table S4. Sensitivity analysis: univariable MR of psychiatric disorders on cognition; Table S5. MR-PRESSO: Univariable MR of psychiatric disorders on cognition; Table S6. PheWAS results of SNPs in SZ; Table S7. PheWAS results of SNPs in MDD; Table S8. PheWAS results of SNPs in ADHD; Table S9. PheWAS results of SNPs in BPD; Table S10. List of SNPs associated with cognition in MR analysis with restricted instruments within oxidative stress-related genes; Table S11. MR analysis with restricted instruments of psychiatric disorders on cognition; Table S12. List of SNPs associated with psychiatric disorders in MR analysis with restricted instruments linked to oxidative stress-related genes expression; Table S13. Sensitivity analysis: MR analysis with restricted conditions of psychiatric disorders on cognition; Table S14. MR-PRESSO: MR analysis with restricted conditions of psychiatric disorders on cognition; Table S15. MR analysis with restricted instruments of cognition on psychiatric disorders. Table S16. Two-step MR analysis of psychiatric disorders on cognition; Table S17. Sensitivity analysis: two-step MR analysis of psychiatric disorders on cognition; Table S18. SMR analysis between cognitive performance and oxidative stress-related genes; Table S19. SMR analysis between SZ and oxidative stress-related genes; Table S20. SMR analysis between MDD and oxidative stress-related genes; Table S21. SMR analysis between BPD and oxidative stress-related genes; Table S22. SMR analysis between ADHD and oxidative stress-related genes.

## Abbreviations

The following abbreviations are used in this manuscript:

8-OHdG	8-hydroxy-2-deoxyguanosine
AD	Alzheimer’s disease
ADHD	attention deficit hyperactivity disorder
AN	anorexia nervosa
ASD	autism spectrum disorder
AXDs	anxiety disorders
BPD	bipolar disorder
CHR	clinical high risk
eQTL	expression quantitative trait locus
GWAS	genome-wide association study
HEIDI	heterogeneity in dependent instrument
IVW	inverse variance weighted
MDA	malondialdehyde
MDD	major depressive disorder
MR	Mendelian randomization
OCD	obsessive–compulsive disorder
PGC	Psychiatric Genetics Consortium
PheWAS	phenome-wide association study
PTSD	post-traumatic stress disorder
SMR	summary data-based MR
SNP	single nucleotide polymorphism
STROBE-MR	strengthening the reporting of observational studies in epidemiology using Mendelian randomization
SZ	schizophrenia
