# Supplementary material for: Causal Impacts of Psychiatric Disorders on Cognition and the Mediating Effect of Oxidative Stress: A Mendelian Randomization Study
Source: Antioxidants (Basel). 2025 Jan 29;14(2):162. doi: 10.3390/antiox14020162 (PMC11852177; doi:10.3390/antiox14020162)
Supplement: Supplementary file 1 [file antioxidants-14-00162-s001.zip › Fig.S2.pdf]

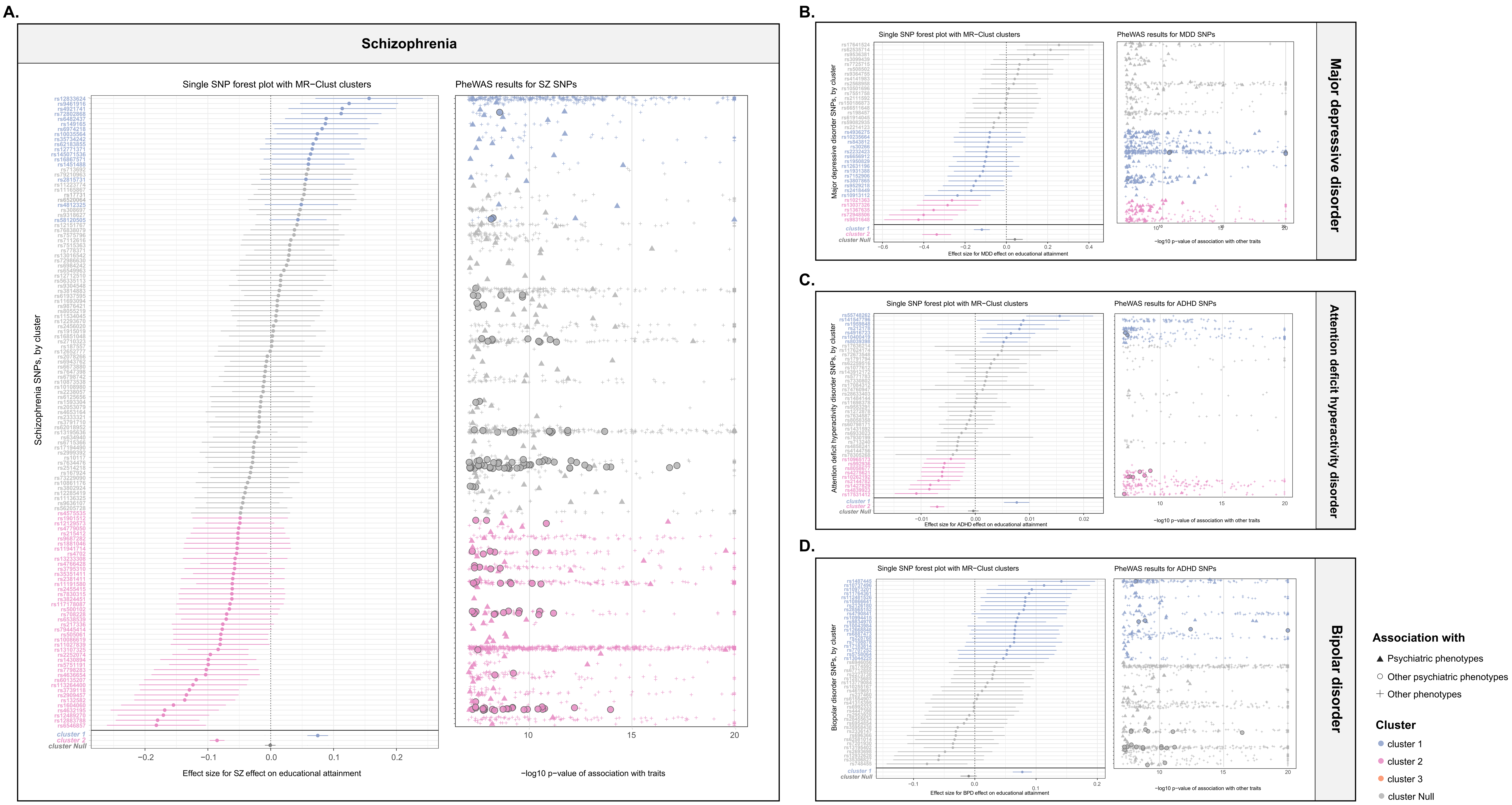
A.
Schizophrenia
Single SNP forest plot with MR-Clust clusters
PheWAS results for SZ SNPs
Schizophrenia SNPs, by cluster
cluster 1
cluster 2
cluster Null
Effect size for SZ effect on educational attainment
-log10 p-value of association with traits
B.
Major depressive disorder
Single SNP forest plot with MR-Clust clusters
PheWAS results for MDD SNPs
Major depressive disorder SNPs, by cluster
Effect size for MDD effect on educational attainment
-log10 p-value of association with other traits
C.
Attention deficit hyperactivity disorder
Single SNP forest plot with MR-Clust clusters
PheWAS results for ADHD SNPs
Attention deficit hyperactivity disorder SNPs, by cluster
Effect size for ADHD effect on educational attainment
-log10 p-value of association with other traits
D.
Bipolar disorder
Single SNP forest plot with MR-Clust clusters
PheWAS results for ADHD SNPs
Bipolar disorder SNPs, by cluster
Effect size for BPD effect on educational attainment
-log10 p-value of association with other traits
Association with
Psychiatric phenotypes
Other psychiatric phenotypes
Other phenotypes
Cluster
cluster 1
cluster 2
cluster 3
cluster Null
