# Supplementary material for: Causal Impacts of Psychiatric Disorders on Cognition and the Mediating Effect of Oxidative Stress: A Mendelian Randomization Study
Source: Antioxidants (Basel). 2025 Jan 29;14(2):162. doi: 10.3390/antiox14020162 (PMC11852177; doi:10.3390/antiox14020162)

Psychiatric disorders	nSNP	β (95% CI)	P value
**Schizophrenia**			
Cognitive performance	13	−0.083 (−0.150, −0.015)	1.70e−02*
Intelligence	14	−0.048 (−0.118, 0.023)	1.83e−01
Educational attainment	17	−0.026 (−0.047, −0.006)	1.11e−02*
**Major depression**			
Cognitive performance	3	−0.317 (−0.521, −0.113)	2.34e−03**
Intelligence	3	−0.325 (−0.523, −0.126)	1.33e−03**
Educational attainment	5	−0.148 (−0.317, 0.021)	8.65e−02
**Bipolar disorder**			
Cognitive performance	4	−0.12 (−0.302, 0.062)	1.95e−01
Intelligence	3	−0.04 (−0.277, 0.197)	7.38e−01
Educational attainment	3	0.011 (−0.040, 0.061)	6.84e−01
**Attention deficit hyperactivity disorder**			
Cognitive performance	2	−0.075 (−0.129, −0.020)	7.37e−03**
Intelligence	2	−0.032 (−0.095, 0.030)	3.14e−01
Educational attainment	2	−0.057 (−0.112, −0.001)	4.49e−02*

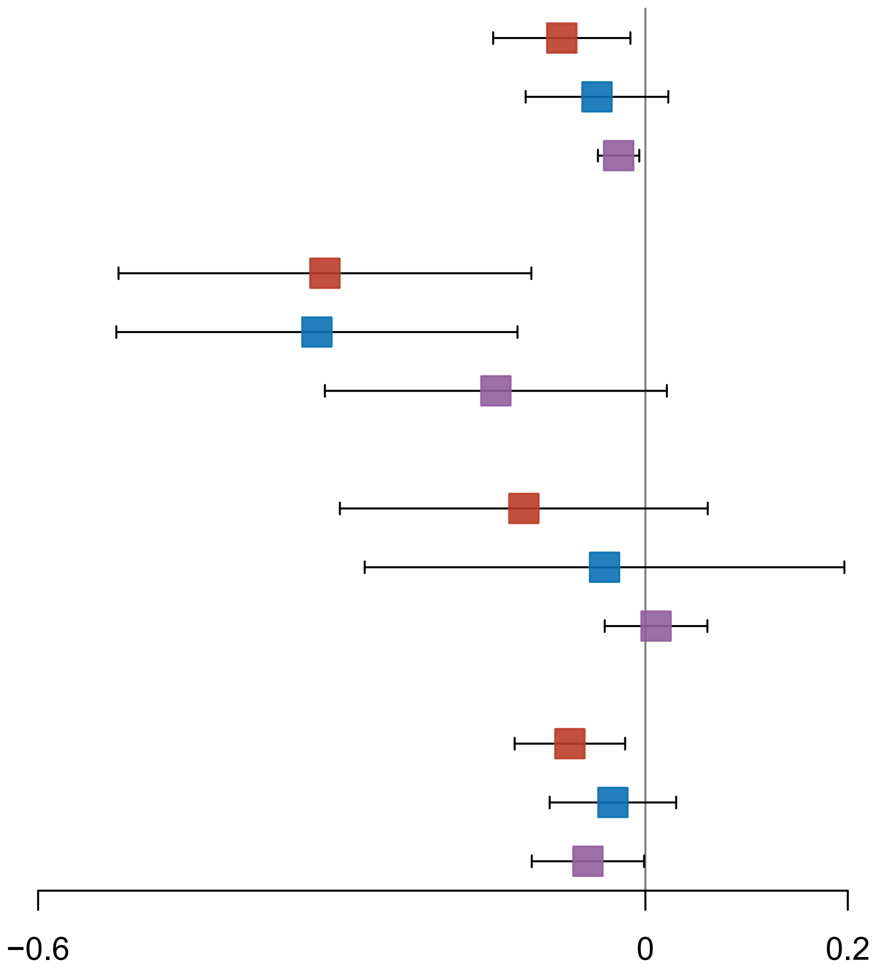

Supplement: Supplementary file 1 [file antioxidants-14-00162-s001.zip › Fig.S3.pdf]
