# Supplementary material for: Causal Impacts of Psychiatric Disorders on Cognition and the Mediating Effect of Oxidative Stress: A Mendelian Randomization Study
Source: Antioxidants (Basel). 2025 Jan 29;14(2):162. doi: 10.3390/antiox14020162 (PMC11852177; doi:10.3390/antiox14020162)
Supplement: Supplementary file 1 [file antioxidants-14-00162-s001.zip › supplemental materials.pdf]

Supplemental materials

Fig. S1 Exploring the heterogeneity of genetic instruments of four psychiatric disorders on intelligence.

Fig. S2 Exploring the heterogeneity of genetic instruments of four psychiatric disorders on educational attainment.

Fig. S3 The estimates of Mendelian randomization analysis with restricted instrumental variables associated with the brain expression of oxidative stress genes.

Fig. S4 Results of Mendelian randomization analysis focusing on the causal effects of oxidative stress traits on psychiatric disorders.

Table S1. Summary of the GWAS Data Used in the MR Analyses.

Table S2. List of oxidative stress-related genes.

Table S3. Univariable MR of psychiatric disorders on cognition.

Table S4. Sensitivity analysis: Univariable MR of psychiatric disorders on cognition.

Table S5. MR-PRESSO: Univariable MR of psychiatric disorders on cognition.

Table S6. PheWAS results of SNPs in SZ.

Table S7. PheWAS results of SNPs in MDD.

Table S8. PheWAS results of SNPs in ADHD.

Table S9. PheWAS results of SNPs in BPD.

Table S10. List of SNPs associated with cognition in MR analysis with restricted instruments within oxidative stress-related genes.

Table S11. MR analysis with restricted instruments of psychiatric disorders on cognition.

Table S12. List of SNPs associated with psychiatric disorders in MR analysis with restricted instruments linked to oxidative stress-related gene expression.

Table S13. Sensitivity analysis: MR analysis with restricted conditions of psychiatric disorders on cognition.

Table S14. MR-PRESSO: MR analysis with restricted conditions of psychiatric disorders on cognition.

Table S15. MR analysis with restricted instruments of cognition on psychiatric disorders.

Table S16. Two-step MR analysis of psychiatric disorders on cognition.

Table S17. Sensitivity analysis: two-step MR analysis of psychiatric disorders on cognition.

Table S18. SMR analysis between cognitive performance and oxidative stress-related genes.

Table S19. SMR analysis between SZ and oxidative stress-related genes.

Table S20. SMR analysis between MDD and oxidative stress-related genes.

Table S21. SMR analysis between BPD and oxidative stress-related genes.

Table S22. SMR analysis between ADHD and oxidative stress-related genes.

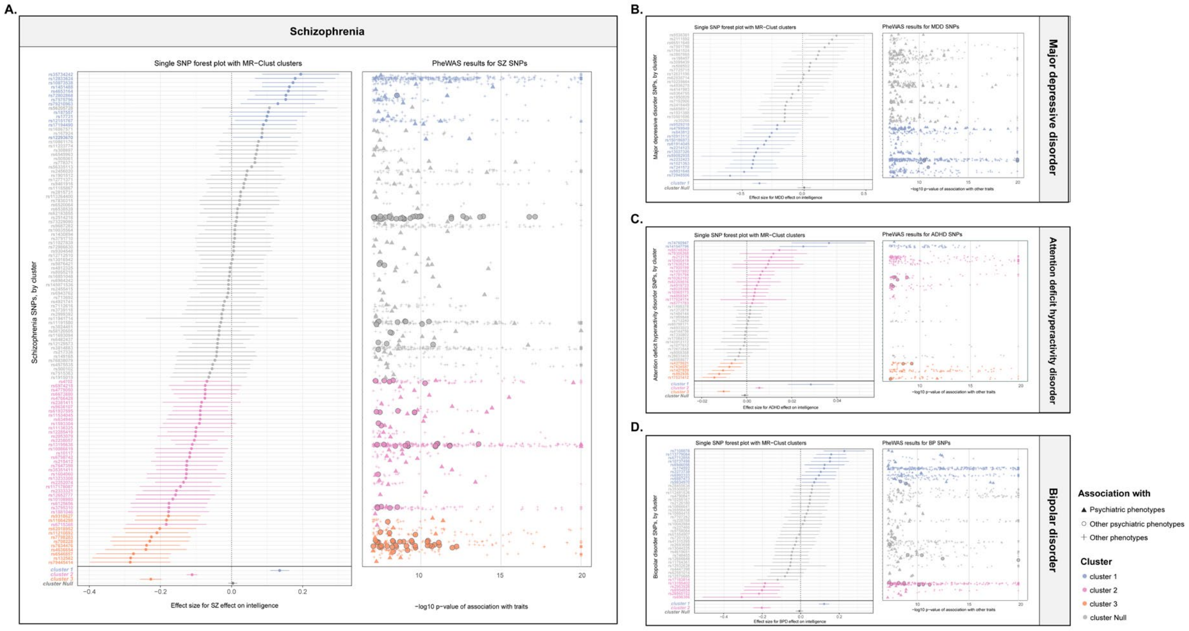

Fig. S1 Exploring the heterogeneity of genetic instruments of four psychiatric disorders on intelligence. A. Left plot is **s**ingle SNP forest plot of schizophrenia on intelligence. SNPs coloured by the cluster membership assigned by MR-Clust. The inverse-variance weighted (IVW) MR estimates for each cluster are presented below single SNP estimates. The error bars are 95% confidence intervals of the Wald Ratio point estimate (beta coeficient) for each variant. **Right plot** is bubble plot of PheWAS results for schizophrenia genetic variants. The data points are other traits associated with SNPs (y-axis) at p-value (FDR-adjusted) < 5×10^{-8} (x-axis, -log10 scale, capped at value 20). **B.** Plots of major depressive disorder on intelligence. **C.** Plots of attention deficit hyperactivity disorder on cognitive performance. **D.** Plots of bipolar disorder on intelligence.

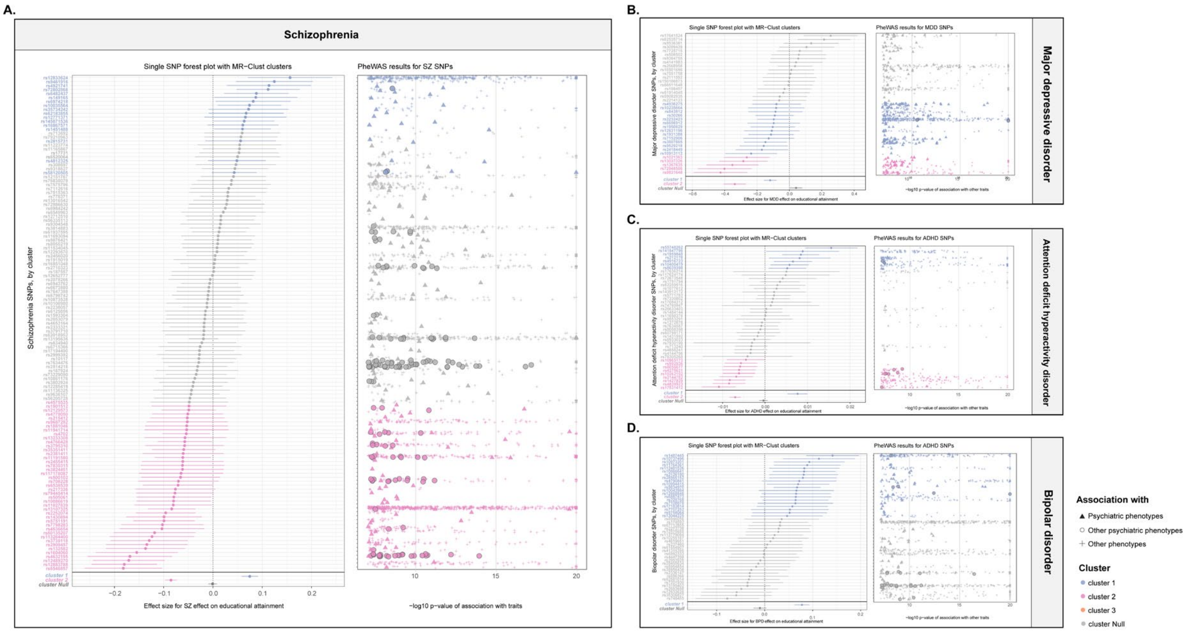

Fig. S2 Exploring the heterogeneity of genetic instruments of four psychiatric disorders on educational attainment.

A. Left plot is **s**ingle SNP forest plot of schizophrenia on educational attainment. SNPs coloured by the cluster membership assigned by MR-Clust. The inverse-variance weighted (IVW) MR estimates for each cluster are presented below single SNP estimates. The error bars are 95% confidence intervals of the Wald Ratio point estimate (beta coeficient) for each variant. **Right plot** is bubble plot of PheWAS results for schizophrenia genetic variants. The data points are other traits associated with SNPs (y-axis) at p-value (FDR-adjusted) $< 5 \times 10^{-8}$ (x-axis, -log10 scale, capped at value 20). **B.** Plots of major depressive disorder on educational attainment. **C.** Plots of attention deficit hyperactivity disorder on cognitive performance. **D.** Plots of bipolar disorder on educational attainment.

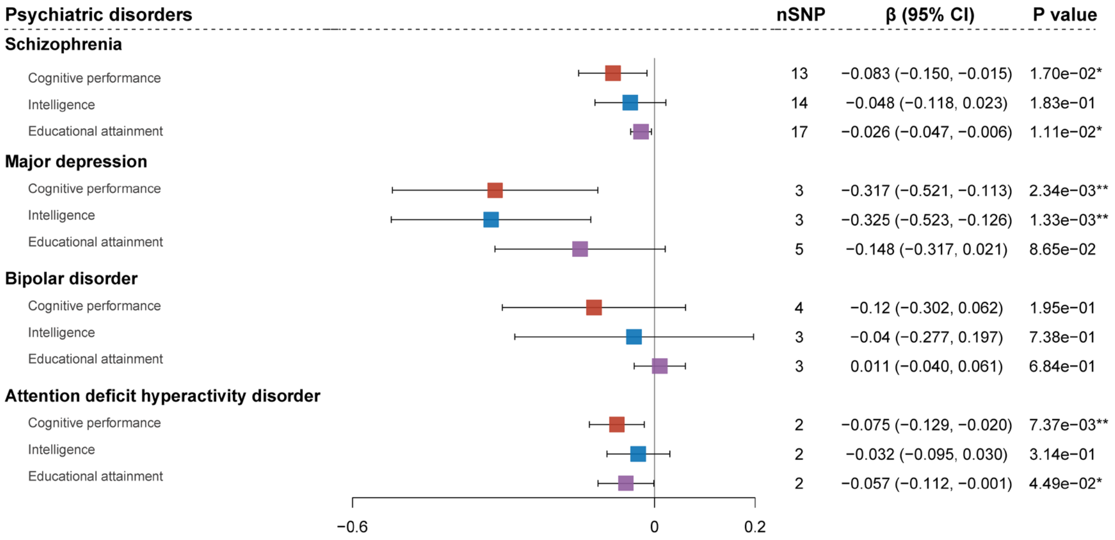

Fig. S3 The estimates of Mendelian randomization analysis with restricted instrumental variables associated with the brain expression of oxidative stress genes. Four psychiatric disorders include schizophrenia (SZ), major depressive disorder (MDD), bipolar disorder (BPD), and attention deficit hyperactivity disorder (ADHD). Three dimensions of cognitive phenotypes include cognitive performance, intelligence, and educational attainment. Plots (bars) represent β (95% CI). Red plots represent the effect of psychiatric disorders on cognitive performance; Blue plots represent the effect of psychiatric disorders on intelligence; Purple plots represent the effect of psychiatric disorders on educational attainment.

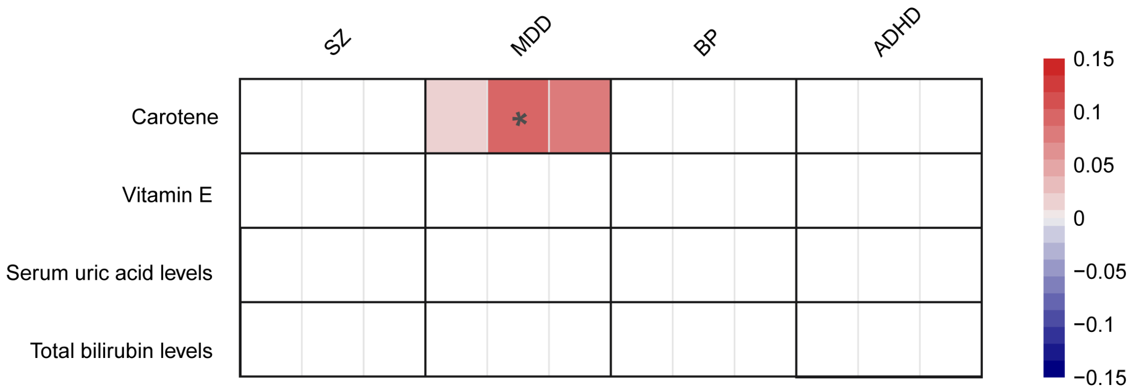

Fig. S4 Results of Mendelian randomization analysis focusing on the causal effects of oxidative stress traits on psychiatric disorders. Psychiatric disorders include schizophrenia (SZ), major depressive disorder (MDD), bipolar disorder (BPD), and attention deficit hyperactivity disorder (ADHD). The plot shows the causal effects of oxidative stress traits (exposure traits) on psychiatric disorders (outcome traits). Coloured cells indicate the point estimate (beta coefficient) from MR analyses, including MR-Egger analyse (left), IVW MR analyse (median) and weight median MR analyse (right).
