# Supplementary figures and images for: Causal Impacts of Psychiatric Disorders on Cognition and the Mediating Effect of Oxidative Stress: A Mendelian Randomization Study
Source: Antioxidants (Basel). 2025 Jan 29;14(2):162. doi: 10.3390/antiox14020162 (PMC11852177; doi:10.3390/antiox14020162)

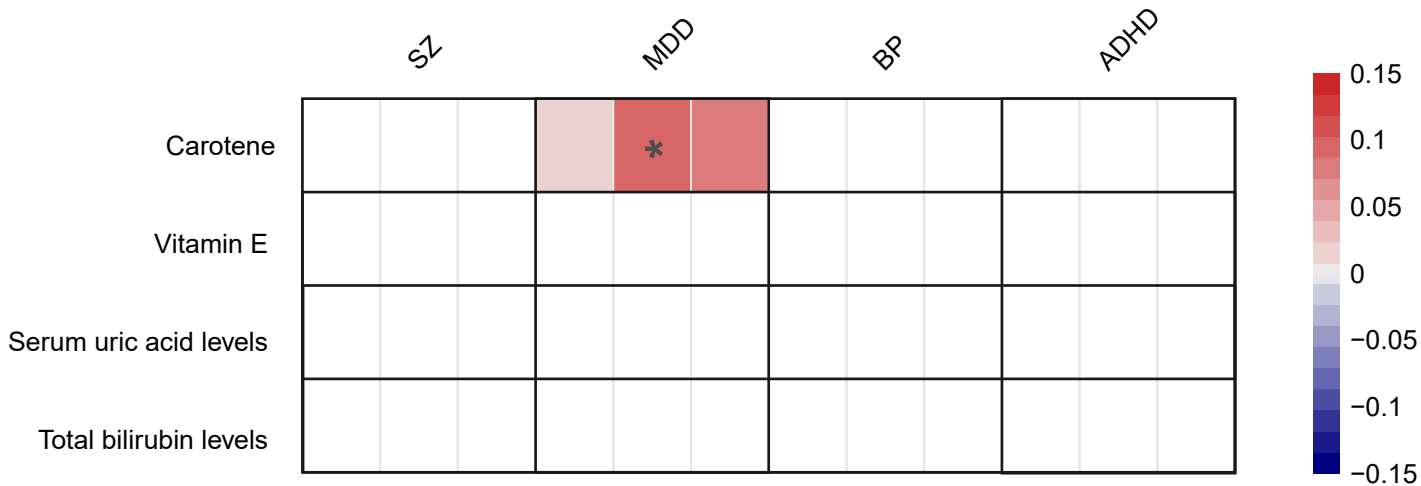

Supplement: Supplementary file 1 [file antioxidants-14-00162-s001.zip › Fig.S4.pdf]
